# PHSNet: A Small-Target Infrared Hotspot Detection Network for Photovoltaic Modules in UAV Remote-Sensing Images

**DOI:** 10.3390/jimaging12060221

**Published:** 2026-05-25

**Authors:** Bingpeng Gao, Yunbo Yang, Xingzhi Chen, Xin Cai, Xinyuan Nan

**Affiliations:** School of Intelligence Science and Technology, Xinjiang University, Urumqi 830017, China; xjugaobp@xju.edu.cn (B.G.); 18853836710@163.com (Y.Y.); chen_xingzhi@xju.edu.cn (X.C.); xynan@xju.edu.cn (X.N.)

**Keywords:** UAV remote-sensing images, photovoltaic (PV) hotspot detection, small target detection, YOLO11n

## Abstract

With the rapid expansion of global photovoltaic (PV) installed capacity, hot spot defects have become a major hidden danger that reduces power generation efficiency and threatens the safe and stable operation of PV stations. Unmanned aerial vehicle (UAV) infrared remote sensing is a key technology for the efficient intelligent monitoring of large-scale PV stations. However, detecting tiny hotspots in such infrared images poses severe challenges. Most of these defects are ultra-small targets with extremely low pixel size and weak contrast, which are easily submerged by complex background noise, leading to prominent issues including low detection accuracy and high miss rates. To address these issues, we propose a lightweight detection network based on YOLO11n, named PHSNet, for PV hotspot detection in UAV infrared images. Its core designs include the dynamic convolution integrated C3k2 (Dy-C3k2) for small target feature enhancement, context-guided downsampling (CG-Down) to alleviate feature loss during downsampling, optimized detection layers, and a lightweight shared deconvolutional detection head (LSDECD) for small target adaptation in low-altitude aerial scenes, forming a full-link optimization architecture for tiny target feature perception. Experiments on a dedicated dataset (4025 images, 25,181 annotations, 92% targets < 20 pixels) show that PHSNet achieves 0.73 AP_50_ and 0.315 AP, surpassing YOLO11n by 0.1 in AP_50_ and 0.058 in AP, respectively. With only 1.8 M parameters and 98.8 FPS, it outperforms mainstream lightweight models, including YOLOv8n and RT-DETR-R18, strikes a superior accuracy–efficiency balance, and provides an efficient solution for real-time intelligent monitoring and edge deployment of PV stations.

## 1. Introduction

Against the backdrop of the global energy transition, photovoltaic energy, as a core component of clean energy, has maintained rapid and continuous growth in its installed capacity [1]. During long-term outdoor operation, PV modules are prone to hotspot defects due to factors such as dust coverage, shading, and cell aging. These defects not only lead to a significant decline in module power generation efficiency but also accelerate module aging and even pose fire risks, seriously threatening the safe and stable operation of PV power plants [2,3]. For example, in April 2025, a rooftop photovoltaic facility of a factory in Huizhou, Guangdong, formed hotspots due to dust accumulation and shading from bird droppings. Combined with the high-humidity and dusty environment that accelerated module aging, these hotspots caused a fire to break out, resulting in damage to the PV system and roof structure. In May 2025, a PV noise barrier on an elevated road in Shanghai generated hotspots due to fallen leaf shading, and the modules were not equipped with module-level shutdown devices, leading to a fire and disrupting elevated traffic for 2 h.

Existing PV module defect detection methods can be roughly divided into traditional image processing-based methods [4] and deep learning-based methods [5]. Traditional methods mainly rely on classical image processing techniques, typically including three stages: preprocessing (e.g., grayscale conversion, filtering, edge detection), handcrafted feature extraction (e.g., texture, shape, frequency domain features), and rule-based classification (e.g., threshold segmentation, contour analysis). For instance, some studies have used Fourier transform [6] and wavelet transform [7] techniques to detect defects such as cracks, black cores, and broken fingers. These methods are low-cost and highly interpretable but suffer from limited detection accuracy, vulnerability to noise interference, insufficient robustness, and poor generalization ability. They struggle to capture hotspot defects, resulting in unsatisfactory performance in PV panel hotspot detection. In contrast, infrared thermography technology has become the mainstream technical means for hotspot defect detection due to its advantages of non-contact and rapid response. With the advancement of UAV infrared remote-sensing technology, UAV-based aerial infrared hotspot detection solutions via remote-sensing images have been widely applied in the intelligent operation and maintenance of large-scale PV power plants [8], owing to their core advantages of wide monitoring coverage and high operational efficiency.

Deep learning-based detection methods have attracted increasing attention due to their powerful feature learning capabilities and environmental adaptability [9,10]. These methods usually rely on large-scale annotated datasets and convolutional neural networks (CNNs) [11] to automatically extract and learn defect-discriminative features. Deep learning detection models can be divided into one-stage and two-stage models: One-stage models such as YOLO (You Only Look Once) [12,13,14,15,16,17,18,19,20] and SSD (Single Shot MultiBox Detector) [21] offer high inference speed and are suitable for real-time detection scenarios; two-stage models such as the R-CNN series [22,23,24,25] typically achieve higher detection accuracy by first generating candidate regions and then completing classification and localization in separate stages. With the increasing application and development of Transformers in natural language processing, Carion et al. [26] proposed DETR (Detection Transformer), which introduced Transformers into object detection tasks. It abandons anchor boxes and non-maximum suppression (NMS) and adopts a one-to-one bipartite matching strategy, realizing an end-to-end detection framework. Zhao et al. [27] proposed RT-DETR (Real-Time Detection Transformer), which not only breaks through the bottleneck of DETR in practicality but also effectively overcomes the performance limitations of one-stage detectors in key links. Compared with traditional methods, deep learning methods exhibit superior performance in infrared thermography, especially in detecting tiny hotspots and suppressing heterogeneous noise, and have become the mainstream approach in modern PV hotspot detection. However, PV hotspot detection in aerial photography scenarios still faces significant technical challenges, mainly due to the extremely small pixel ratio of hotspot targets, as well as problems such as low contrast and complex backgrounds. These issues lead to traditional object detection models being prone to loss of subtle features, high missed detection rates, and insufficient detection accuracy. Therefore, timely and accurate detection of PV module hotspot defects is crucial for ensuring the operation and maintenance efficiency and safety of power plants.

In response to challenges in the detection of PV module hotspots, scholars at home and abroad have conducted extensive research. Peng et al. [28] addressed the problems of complex defect backgrounds and small target sizes in infrared images of PV cells, proposing the FSC-Net model improved from RT-DETR-R18. By optimizing the backbone and neck network structures and introducing the Inner-WIoUv3 loss function, they achieved simultaneous improvements in model lightweighting and defect detection accuracy. Cao et al. [29] tackled the common issues of difficulty in recognizing small targets and high computational cost of detection algorithms during airborne infrared imaging-based PV hotspot detection, proposing a lightweight Transformer-based PV hotspot detection algorithm. Through the design of hierarchical instance enhancement, wavelet attention enhancement, and multi-scale feature fusion modules, they significantly reduced computational overhead while ensuring high detection accuracy. However, compared with YOLO series models, the improved Transformers used in both studies still face the problem of high model complexity. Liu et al. [30] aimed at the low detection accuracy of small-target hotspot defects in infrared images of PV modules and redundant model parameters, proposing an improved method based on YOLOv8n. By reconstructing the backbone network, designing the DCS-SE attention mechanism, and adopting the STAW-IoU loss function, they achieved the unification of model lightweighting, improved detection accuracy, and good generalization performance. Hao et al. [31] addressed the insufficient feature expression caused by diverse defect shapes and environmental interference when thermal infrared sensors detect PV hotspot faults, proposing an adaptive detection network based on region perception and cross-channel feature aggregation. Through collaborative optimization of multiple modules, they achieved high hotspot detection accuracy in complex detection environments. However, neither of these studies considered extremely small-pixel targets and dense hotspot distribution scenarios, resulting in certain limitations. To address the challenges of complex thermal characteristics, environmental noises, and difficulties in locating tiny faults in thermal infrared images of photovoltaic (PV) panels, Awedat et al. [32] proposed an enhanced U-Net architecture integrating residual blocks, atrous spatial pyramid pooling (ASPP), and attention mechanisms. This architecture significantly improves fault segmentation accuracy and reduces false positive rates, but it is not suitable for complex backgrounds.

To address the core challenges in small infrared hotspot target detection of PV modules in UAV aerial photography scenarios, including easy feature loss of ultra-small targets, low detection accuracy, high miss rates, and the difficulty in achieving collaborative optimization between model lightweight design and detection performance, this paper presents PHSNet, a lightweight improved detection model based on YOLO11n, which is a strong and well-optimized combination of existing mature modules in four aspects, including feature enhancement, feature preservation, scale adaptation, and lightweight design. Compared with the YOLO11n and previous small-target detection models, the core innovations lie in the optimization for ultra-small targets (<20 pixels) in UAV infrared PV scenes, which breaks through the limitations of YOLO11n’s structure and the single-module optimization of existing lightweight models. The main improvements are summarized as follows:

(1) The C3k2 module employs static convolutional kernels, which have limited adaptive receptive field adjustment for tiny and low-contrast targets. In this paper, a Dy C3k2 module is designed by introducing dynamic convolution into the C3k2 bottleneck structure. Through attention-based weighting, multiple prototype convolution kernels are adaptively fused to enhance the feature capture capability for low-contrast ultra-small photovoltaic hot spots.
(2) The native downsampling approach inevitably loses fine-grained features of ultra-small targets. To address this, a CG Down downsampling module is proposed, which integrates a multi-branch context-aware structure with a dual residual mechanism. During resolution compression, it simultaneously fuses global semantics and multi-scale features, structurally alleviating the information loss of tiny targets during the downsampling process.
(3) The P3–P5 detection head architecture is designed based on the generic object scales of the COCO dataset, which is severely mismatched with the extreme distribution of the dataset in this paper, where 92% of the targets are smaller than 20 pixels. Accordingly, the detection layer configuration is restructured by adding high-resolution P1 and P2 detection layers and removing the redundant P5 layer designed for large objects. This design precisely matches the scale characteristics of photovoltaic hot spots and maximally preserves the details of tiny targets.
(4) The native detection head uses batch normalization and standard convolution, which suffers from instability under small batch training and easily loses the weak edge features of ultra-small targets. To tackle these issues, a lightweight LSDECD detection head is proposed. It replaces batch normalization with group normalization to accommodate small batch training, and integrates detail-enhanced convolution (DEConv) with a parameter-sharing strategy, for strengthening the capture of weak edge information and controlling the parameter count.

The rest of this paper is organized as follows. Section 2 elaborates on the overall architecture of PHSNet and the design principles of its core modules. Section 3 introduces the experimental dataset, hardware and software environment, and evaluation metrics, and conducts systematic analysis and verification on the experimental results. Finally, the discussion and conclusions are presented.

## 2. Methods

YOLO11 is the 11th-generation object detection model iterated by Ultralytics based on YOLOv8, and its core improvements are reflected in three aspects. First, the backbone and neck networks are reconstructed by replacing the original C2f module with the C3K2 module and adding the C2PSA module, which enhances feature extraction capability and detection accuracy while reducing the number of parameters and computational complexity. Second, the training pipeline is optimized to improve image processing efficiency and accelerate model training and convergence speed. Third, it supports multi-platform deployment on cloud platforms, edge devices, and mobile devices.

**Figure 1:**
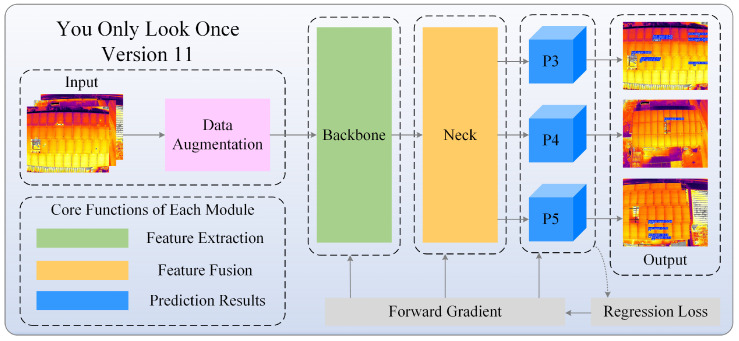
Network structure of YOLO11.

YOLO11 inherits the three-level network architecture of YOLOv8. The backbone network, composed of Conv, C3k2, SPPF, C2PSA and other modules, is responsible for extracting core features of the input image. The neck adopts the PANet architecture to achieve deep fusion of multi-scale features. The head is equipped with three detection heads of different sizes, which are adapted to the detection tasks of small, medium and large objects, respectively, and output the category, coordinates and confidence information of the targets. The model includes five scaled versions: YOLO11n, YOLO11s, YOLO11m, YOLO11L and YOLO11x. Among them, YOLO11n achieves a favorable balance between accuracy and speed with a lightweight architecture, so it is selected as the baseline model in this study [33]. The network structure of YOLO11 is shown in Figure 1.

**Figure 2:**
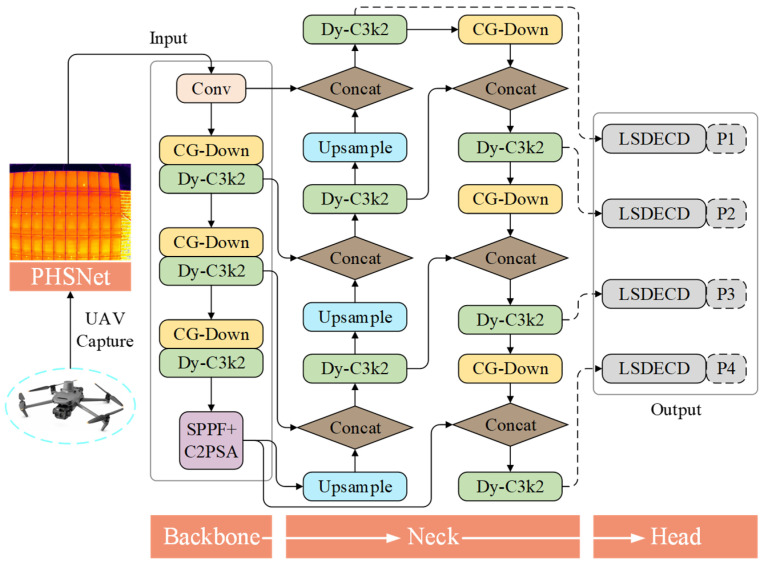
Network structure of PHSNet.

Although YOLO11 improves the detection performance of general objects through structural optimization, it still has limitations in the detection of extremely small objects such as aerial photovoltaic hotspots. The traditional downsampling operation in its backbone network easily leads to the loss of subtle hotspot features, and the original multi-scale detection heads have insufficient adaptability to hotspot targets with an extremely small pixel proportion in aerial scenarios, which directly restricts the improvement of detection accuracy. To realize efficient and accurate identification of hotspots in aerial photovoltaic module images, based on the YOLO11n detection framework, this study proposes an infrared hotspot detection algorithm for photovoltaic modules, named PHSNet, which integrates the dynamic convolution module, context-guided module, LSDECD lightweight detection head, and the optimization of detection layer scale and quantity. The overall algorithm structure, as shown in Figure 2, is mainly composed of a backbone feature extraction network, a neck network and a multi-scale detection head, undertaking feature modeling tasks at different levels, respectively.

### 2.1. Dy-C3k2 Feature Extraction Module

**Figure 3:**
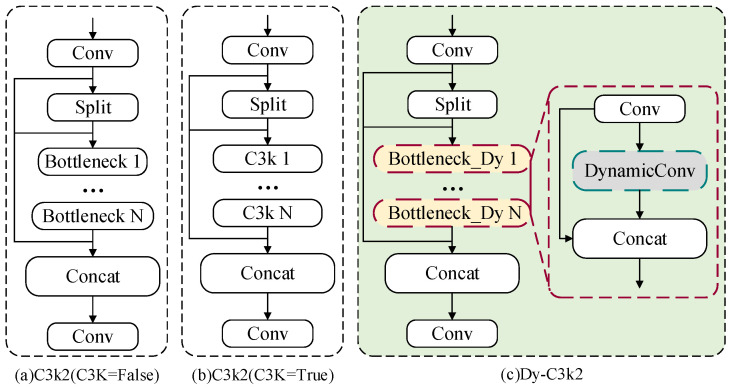
Structural comparison of C3k2 variants and the proposed Dy-C3k2. (**a**) Standard C3k2 (C3K = False) with vanilla Bottlenecks; (**b**) C3k2 (C3K = True) with C3k blocks; (**c**) Dy-C3k2 with Bottleneck_Dy blocks, whose internal structure (including DynamicConv) is shown in the red dashed box. N is the number of stacked blocks.

As a core feature extraction component in YOLO11, the C3k2 module exhibits inherent limitations in adaptive receptive field adjustment and complex contextual information capture owing to its static convolution kernel structure when facing tiny targets and low-contrast targets, thereby leading to insufficient representation ability for subtle features. To alleviate this problem, this study introduces the dynamic convolution unit [34] to enhance the detection performance for tiny and low-contrast targets, and the improved C3k2 module is denoted as Dy-C3k2.The structural comparison between C3k2 and Dy-C3k2 is illustrated in Figure 3.

Dynamic convolution predefines a set of K prototype convolution kernels $\left\{\left.{\mathrm{conv}}_{1},{\mathrm{conv}}_{2},\ldots ,{\mathrm{conv}}_{k}\right\}\right.$, and then employs a lightweight attention network to dynamically generate the corresponding weight coefficient $\pi_{k}(x)$ for each prototype convolution kernel based on the input feature x. By performing weighted fusion of all prototype convolution kernels using these coefficients, dynamic convolution can adaptively adjust the convolution kernel parameters according to the distribution characteristics of the input feature x, ultimately generating a dedicated convolution kernel tailored to the current input. The specific mathematical expressions of the above process are given in Equations (1)–(3).

(1) $$W_{\mathrm{dyn}}(x)=\sum_{k=1}^{K}\pi_{k}(x)\;W^{\left(k\right)}$$

(2) $$b_{\mathrm{dyn}}(x)=\sum_{k=1}^{K}\pi_{k}(x)\;b^{\left(k\right)}$$

(3) $$y=W_{\mathrm{dyn}}(x)*x+b_{\mathrm{dyn}}(x)$$

where $W_{\mathrm{dyn}}(x)$ denotes the adaptive convolution kernel matrix dynamically generated based on the input feature x; K is the preset total number of base convolution kernels; $\pi_{k}(x)$ represents the attention weight coefficient associated with the input feature x; $W^{\left(k\right)}$ corresponds to the K-th base convolution kernel matrix; $b_{\mathrm{dyn}}(x)$ is the dynamically generated bias vector; and $b^{\left(k\right)}$ is the K-th base bias vector.

**Figure 4:**
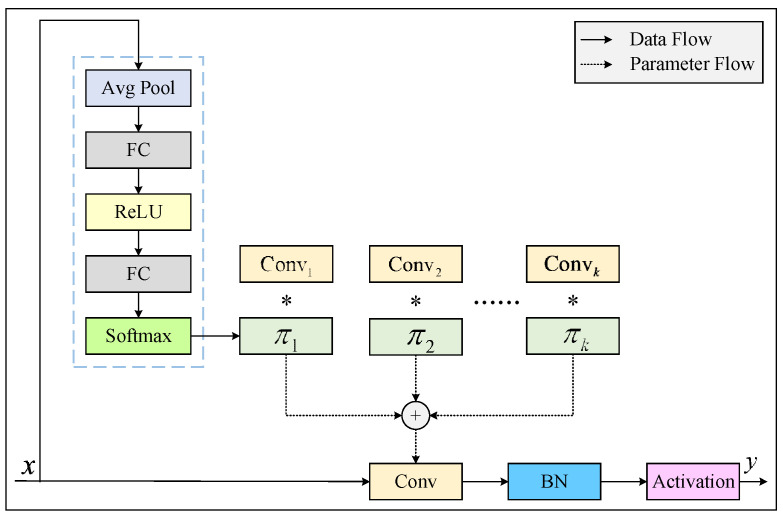
Overview of the proposed dynamic convolution module. Solid arrows denote data flow, and dashed arrows denote parameter flow.

The attention weight coefficient $\pi_{k}(x)$ is adaptively generated by the lightweight attention network shown in the blue dashed box of Figure 4, and its specific generation process is as follows. First, average pooling is performed on the input feature map x to aggregate spatial dimension information and extract the global feature description vector z. Subsequently, this global feature description vector z is fed into a mapping network composed of two fully connected layers, where the intermediate layer realizes nonlinear feature transformation via the ReLU activation function. Finally, normalization is conducted through the Softmax function to output the attention weight coefficient $\pi_{k}(x)$ that satisfies the constraint that the sum of weights equals 1. The generation process of the above attention weights can be mathematically characterized by Equations (4)–(6).

(4) $$z_{c}=\frac{1}{HW}\sum_{i=1}^{H}\sum_{j=1}^{W}x_{c,i,j},z\in \;{\mathbb{R}}^{C_{\mathrm{in}}}$$

(5) $$s=W_{2}\mathrm{ReLU}\;(W_{1}z+b_{1})+b_{2},s\in {\mathbb{R}}^{K}$$

(6) $$\pi_{k}(x)=\frac{\exp(s_{k})}{\sum_{k^{\prime }=1}^{K}\exp(s_{k\prime })},\pi (x)\in {\mathbb{R}}^{K}$$

where $z_{c}$ denotes the output result after applying average pooling to the c-th channel of the input feature map; H and W correspond to the height and width dimensions of the input feature map, respectively; $x_{c,i,j}$ represents the feature value at the spatial position (i,j) of the c-th channel in the input feature map; $C_{in}$ denotes the total number of channels of the input feature map; $W_{1}$ and $W_{2}$ are the weight vectors of the two fully connected layers in the attention network, respectively, and $b_{1}$ $b_{2}$ are the bias vectors of the corresponding fully connected layers; s is the intermediate feature vector output by the fully connected layers, and K denotes the dimension size of this intermediate feature vector s; $s_{k}$ represents the feature score of the k-th branch, and $s_{k^{\prime }}$ represents the feature score of the $k^{\prime }$-th branch.

Unlike traditional static convolution that employs fixed convolution kernels, dynamic convolution can dynamically select and fuse multiple convolution kernels based on the input features, thereby adaptively capturing the complex contextual information of each input feature and the intricate relationships between targets and the background, and effectively supplementing the critical global contextual information that is often absent in local multi-scale features. Dy-C3k2 integrates dynamic convolution into the bottleneck layer of the backbone C3k2 module. This integration facilitates the stacking and continuous fusion of small-target features, enriches the contextual information extracted from the input data by deepening the network structure, and enhances the ability to separate targets from the background—particularly improving the detection performance of tiny targets in similar backgrounds.

### 2.2. CG-Down Downsampling Module

In the task of infrared hotspot detection for PV modules, the CG-Down module is constructed by integrating the context-guided block (CG Block) [35] into the downsampling procedure. Unlike traditional downsampling operations, which tend to lose the fine-grained features of tiny targets, the core advantage of this module lies in its ability to actively capture and fuse global contextual semantics and multi-scale features. It enables the network, during resolution compression, to not only focus on local details but also understand the correlations between hotspots, the background of the whole PV module, and adjacent normal regions. In this way, the network’s ability to perceive and preserve features of small and blurred hotspots is strengthened, and the interference from complex backgrounds is effectively suppressed.

**Figure 5:**
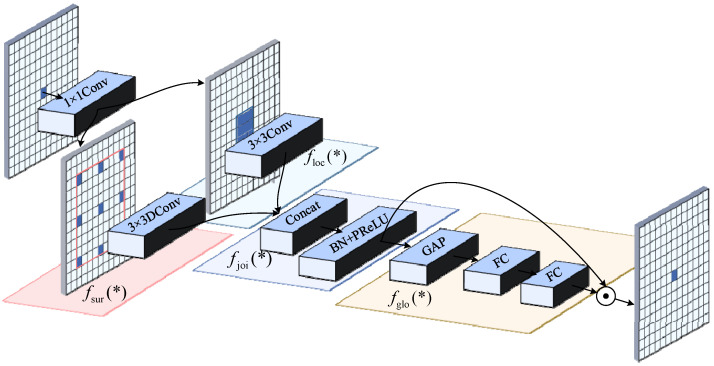
Network structure of CG Block.

The CG Block is a feature enhancement module tailored for computer vision tasks. Its core goal is to fully mine and fuse local detailed features, surrounding contextual features, and global semantic features, so as to alleviate the insufficient perception of complex scenes by conventional feature extraction modules. Meanwhile, it optimizes the training process through residual learning, ultimately improving the model’s feature representation capability and task performance. As illustrated in Figure 5, this module consists of four components: a local feature extractor $f_{\mathrm{loc}}(*)$, a surrounding context extractor $f_{\mathrm{sur}}(*)$, a joint feature extractor $f_{\mathrm{joi}}(*)$, and a global context extractor $f_{\mathrm{glo}}(*)$.

The input of the CG Block is the feature map X. First, feature preprocessing is performed to compress the number of channels and reduce subsequent computational cost. Second, local detailed features and surrounding contextual features are extracted via two independent parallel paths, respectively, and this process is mathematically formulated in Equations (7)–(9).

(7) $$X_{\mathrm{pre}}={\mathrm{Conv}}_{1\times 1}(X)$$

(8) $$L=f_{\mathrm{loc}}(X_{\mathrm{pre}})={\mathrm{Conv}}_{3\times 3}(X_{\mathrm{pre}})$$

(9) $$S=f_{\mathrm{sur}}(X_{\mathrm{pre}})={\mathrm{DilatedConv}}_{3\times 3}(X_{\mathrm{pre}})$$

After obtaining the local feature L and surrounding contextual feature S, the module fuses them via channel concatenation, followed by batch normalization (BN) and parametric ReLU (PReLU) activation to yield the joint feature J. This process can be formally defined in Equation (10).

(10) $$J=f_{\mathrm{joi}}(L,S)=\mathrm{PReLU}(\mathrm{BN}(\mathrm{Concat}(L,S)))$$

The global context extractor applies global average pooling (GAP) to the joint feature J to obtain the global context vector. This vector is then fed into two fully connected (FC) layers and a Sigmoid function sequentially to generate channel attention weights W ranging from 0 to 1. Finally, feature recalibration is achieved by element-wise channel multiplication between W and J. This process is described in Equations (11)–(13).

(11) G=GAP(J)

(12) $$W=\sigma ({\mathrm{FC}}_{2}(\mathrm{ReLU}({\mathrm{FC}}_{1}(G))))$$

(13) $$Y=f_{\mathrm{glo}}(J,W)=J⊙W$$

where σ denotes the Sigmoid function, and ⊙ represents element-wise channel multiplication. The output Y is the final feature map enhanced by context guidance.

**Figure 6:**
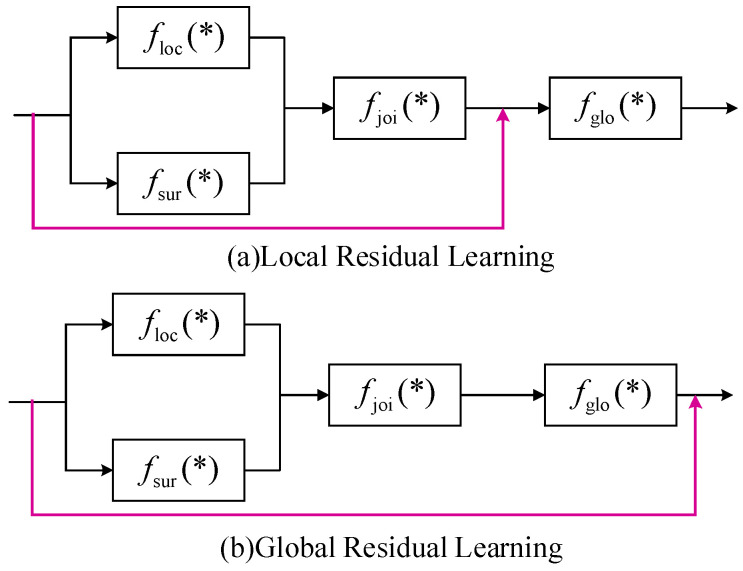
Structure of local residual learning (LRL) and global residual learning (GRL).

By leveraging the residual learning mechanism, the CG Block can effectively adapt to complex environmental features and optimize the gradient backpropagation process during the training phase. As illustrated in Figure 6, two residual connection structures are designed in this module. The first is local residual learning (LRL), which fuses the input features with the features learned by the joint feature extractor. The second is global residual learning (GRL), which integrates the input features with the features acquired by the global context extractor. Compared with LRL, GRL demonstrates superior performance in facilitating information propagation within the network [36].

### 2.3. Optimization of the Number and Scale of Detection Layers

In the hotspot detection task for photovoltaic modules in this study, the detection head, as a core component of the object detection model, undertakes the final responsibility of decoding feature maps and outputting target class probabilities and precise bounding box coordinates. The baseline model YOLO11n adopts a multi-scale detection mechanism, whose three native detection heads correspond to feature map resolutions of 80 × 80, 40 × 40, and 20 × 20, respectively, aiming to detect objects of small, medium, and large scales. This scale division is generally based on the criteria of the COCO dataset, where 32 × 32 pixels and 96 × 96 pixels are used as the area thresholds for small and medium objects, respectively.

However, the dataset adopted in this study shows an extremely prominent small-object bias. Statistical analysis reveals that annotated instances with bounding box sizes smaller than 20 pixels account for 92% of the total (23,220 instances), whereas those larger than 20 pixels account for only 8%. This implies that the actual sizes of most hotspot targets in the dataset are far smaller than the lower bound of small objects defined by the COCO dataset, presenting a unique detection challenge. To tackle this challenge, targeted optimizations are conducted on the detection head structure in this study. The core improvement lies in significantly enhancing the model’s perception capability for ultra-small objects: on the basis of the original detection layers, an additional high-resolution (160 × 160) P2 detection layer and an ultra-high-resolution (320 × 320) P1 detection layer are incorporated. The fundamental principle of this design is to preserve the detailed information of small objects on the feature maps to the greatest extent by reducing the downsampling factor. Specifically, when the input image size is 640 × 640 pixels, the corresponding perception regions on the original image differ substantially among different detection layers, as listed in Table 1. Such progressively increasing spatial resolution can effectively alleviate the risk that the weak feature signals of tiny hotspots are diluted or overwhelmed by background noise during forward propagation, thereby strengthening the model’s ability to capture critical information.

Meanwhile, considering the extremely low proportion of large-scale objects in the dataset, the original 20 × 20 detection layer designed for large-object detection is underutilized. To improve model efficiency and structural compactness, this P5 detection layer is removed in this study.

### 2.4. Design of the LSDECD Lightweight Detection Head

In the original detection head architecture of YOLO11, data normalization serves as a key component for ensuring model performance, whose core functions lie in accelerating training convergence, enhancing generalization capability, and effectively mitigating issues such as vanishing or exploding gradients. As a mainstream normalization technique, batch normalization (BN) [37] offers advantages including fast convergence speed, low dependence on initial weights, and controllable overfitting risk. However, this method is highly sensitive to the batch size. For the small-batch training scenario with a batch size of 4 adopted in this study, the mean and variance estimated by BN cannot fully characterize the overall data distribution, which easily leads to model performance degradation.

**Figure 7:**
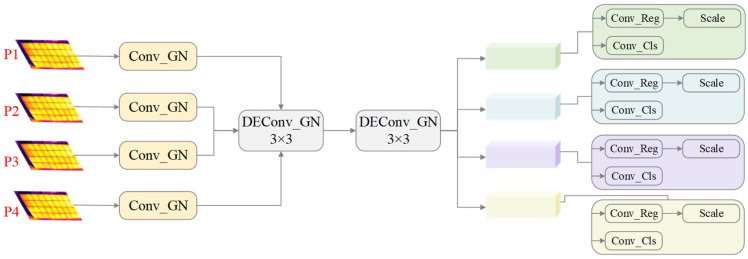
Detailed network structure of LSDECD.

To address this bottleneck, this study proposes a lightweight shared deconvolutional detection head (LSDECD), whose structure is illustrated in Figure 7. Instead of the conventional BN, the proposed detection head adopts group normalization (GN) [38]. By leveraging the core mechanism of channel-wise normalization, it significantly improves the model stability in small-batch training scenarios while optimizing object localization accuracy and classification performance. Furthermore, the newly added P1 and P2 high-resolution detection layers focus on ultra-small hotspot targets, and their feature maps contain a large amount of edge and gradient information of tiny hotspots. However, the original vanilla convolution structure in the detection head has insufficient ability to capture such low-contrast, weak-edge features of tiny targets, and tends to lose critical edge gradient information, which further limits the improvement of small target detection accuracy.

**Figure 8:**
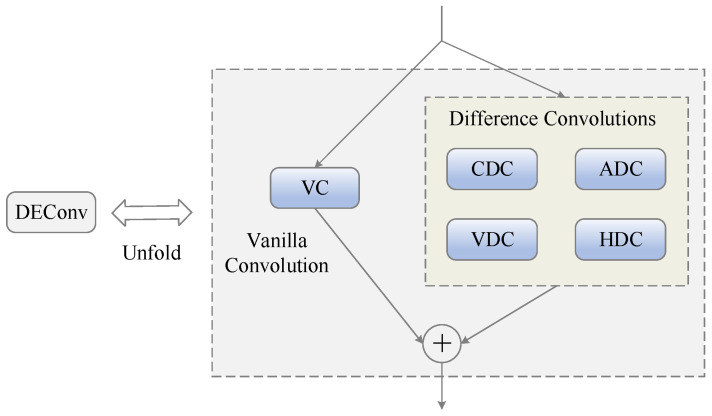
Mechanism of DEConv.

To this end, LSDECD incorporates Detail-Enhanced Convolution (DEConv) into the shared convolutional structure, with its overall architecture illustrated in Figure 8. DEConv consists of five parallel branches, including one Vanilla Convolution (VC) branch and four Differential Convolution (DC) branches, namely Central DC, Angular DC, Horizontal DC, and Vertical DC. The core function of the DC branches is to encode the multi-directional gradient prior of the image and enhance the capture ability of weak edge features.

**Figure 9:**
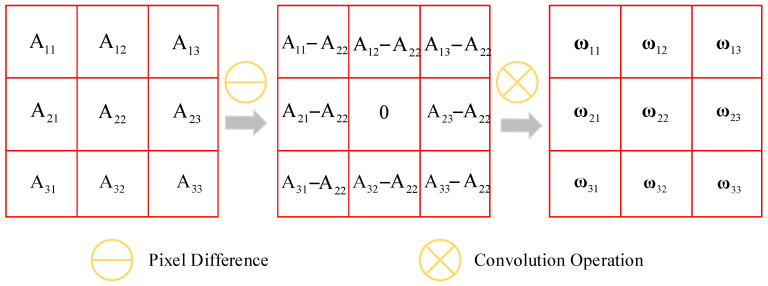
The schematic diagram of central DC.

The core idea of differential convolution is to encode the gradient prior information of traditional local descriptors into the design of the convolution kernel, realize differential operation by reconstructing the weight distribution of the traditional convolution kernel, and then perform convolution operation on the feature map with the differential convolution kernel to directly output the feature map containing gradient-level information without additional post-processing steps, which can significantly improve the feature representation capability and generalization ability of the convolutional layer. The schematic diagram of the calculation principle of Central DC is shown in Figure 9: first, in the local region of the feature map corresponding to the convolution kernel, a differential operator is constructed by calculating the relative difference between the surrounding pixels and the central pixel; then, the differential operator is fused with the convolution weights, and convolutional encoding is performed on the local feature map. The core principle of the other three types of differential convolutions is also based on the relative pixel difference, but there is a clear distinction in the direction of gradient calculation: Angular DC calculates the difference between adjacent pixels in a specific diagonal direction, while Horizontal DC and Vertical DC traverse the feature map along the vertical and horizontal directions, respectively, to calculate the pixel gradients in the corresponding horizontal and vertical directions. This design introduces gradient information from multiple directions into traditional convolution, which accurately compensates for the defect that traditional convolution is prone to losing edge information of tiny targets, and can efficiently capture the weak edge and gradient features of ultra-small PV hotspots.

Meanwhile, considering that the introduction of P1 and P2 detection layers may cause a surge in the number of parameters, LSDECD integrates the original four feature extraction operations into a single pipeline through a parameter-sharing strategy. Combined with the reparameterization characteristic of DEConv, the multi-branch parallel convolution can be equivalently merged into a single standard convolution during the inference phase without introducing additional computational overhead. In addition, a scale adjustment module is employed to match the dimensions of feature maps with different scales, thereby effectively reducing the parameter scale and computational overhead of the detection head.

## 3. Results

### 3.1. Datasets

The raw UAV infrared remote-sensing data used in this study were acquired by an infrared thermal imaging sensor mounted on a DJI Mavic 3T small UAV. The original images are in 16-bit R-JPEG format with a spatial resolution of 640 × 512 pixels. After data screening and quality control, a dedicated UAV infrared remote-sensing dataset for PV module hotspot detection is constructed, comprising 4025 valid images. All images were annotated with the “hotspot” defect category, yielding a total of 25,181 bounding boxes [39].

**Figure 10:**
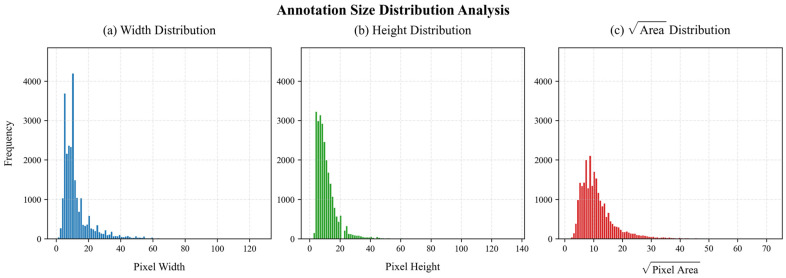
Histogram of bounding box target size distribution.

To comprehensively characterize the scale distribution of the annotated hotspots, three complementary metrics including pixel width, pixel height, and the square root of the bounding box area are analyzed, as shown in Figure 10. Figure 10a,b present the distributions of pixel width and pixel height, respectively. Both of them exhibit a pronounced right-skewed pattern, with the vast majority of bounding boxes concentrated in the 0–20 pixel range, which indicates that the annotated hotspots are predominantly small in size. Notably, the width and height distributions show nearly identical shapes, confirming that the bounding boxes have no extreme aspect ratios, and the limitations of single-dimension scale division are avoided. For this reason, the square root of the bounding box area is adopted as the criterion for absolute scale division to provide a balanced representation of the overall target size. As shown in Figure 10c, the distribution of the square root of the area is also strongly right-skewed, with its peak falling in the 5–10 pixel range. About 91% of the bounding boxes have a square root of area less than 20 pixels, which confirms that the dataset represents a typical small object detection scenario. This scale distribution highlights the core challenge of the task, which is accurate detection of tiny hotspots, and provides clear data-driven support for the subsequent design and optimization of the model’s small object detection capabilities.

The dataset was randomly divided into a training set (2817 images), a validation set (805 images), and a test set (403 images) at a ratio of 7:2:1. Given that ultra-small hotspot detection is prone to overfitting and highly sensitive to hyperparameters, a 20% validation set can improve the reliability of hyperparameter tuning and convergence monitoring, and reduce randomness in model training. The 70% training set ensures sufficient samples for feature learning, and the 10% test set guarantees unbiased and authentic performance evaluation. This partitioning achieves an optimal balance among training, validation and test sets. To meet the input size requirements of the YOLO object detection network, during the training phase, all images were processed using the aspect ratio-preserving LetterBox method. Specifically, after equal-proportion scaling, the images were padded to 640 × 640 pixels with gray (114, 114, 114) pixels to minimize the interference of image deformation on hotspot target features.

### 3.2. Experimental Environment and Parameter Setting

The hardware and software configurations of this experiment are as follows. The experimental platform is built on the Windows 10 operating system, and the computing hardware utilizes an NVIDIA GeForce RTX 4060 Ti GPU and an Intel Core i5-12600KF CPU. The programming language is Python 3.9.21, the deep learning framework is PyTorch 2.5.1, and the CUDA 11.3 toolkit is integrated to enable GPU-accelerated training. The detailed experimental parameter settings are presented in Table 2.

To ensure the fairness of comparative experiments, all YOLO-series models including YOLOv8n, YOLOv9-Tiny, YOLOv10n, YOLO11n, and our proposed PHSNet adopt completely identical training configurations. Meanwhile, the Transformer-based RT-DETR-R18 has slight differences in configuration due to its inherent architectural characteristics, which is a common practice in the fair comparison of different detection frameworks. RT-DETR-R18 adopts its official recommended optimizer (AdamW) and dedicated detection loss functions. Since the transformer architecture relies on specific optimization strategies and loss mechanisms to ensure normal convergence, unifying its configuration with YOLO-series models may lead to training failure and unreliable results, which is not conducive to objective performance comparison.

All quantitative experimental results in this section were obtained from three independent trainings with different random seeds to ensure the reliability of the experimental results.

### 3.3. Model Evaluation Metrics

The confusion matrix is one of the core tools for performance evaluation of classification models and has been widely applied in the quantitative analysis of evaluation metrics in the field of image recognition. In this study, key performance metrics for image recognition tasks are systematically calculated based on the confusion matrix, including core evaluation criteria such as recall (R), precision (P), and average precision (AP). Specifically, AP_50_ refers to the average precision at an Intersection over Union (IoU) threshold of 0.5, reflecting the model’s detection capability for hotspot targets under coarse matching scenarios. AP_50:95_ (i.e., AP) denotes the average precision across IoU thresholds from 0.5 to 0.95 (with a step of 0.05, totaling 10 IoU thresholds), which comprehensively measures the model’s detection completeness and localization accuracy for hotspot targets. To comprehensively evaluate the lightweight level and engineering practicality of the model, lightweight is defined as a multi-dimensional evaluation system in this paper. The static core indicators include parameters and model file size, and the dynamic efficiency indicators include inference speed (FPS), inference latency, and GPU memory usage. Among them, parameters and model size are the primary criteria for measuring lightweight, and dynamic indicators are used as auxiliary verification to judge whether the model meets the deployment requirements of UAV edge devices.

### 3.4. Exploration on Optimization of Detection Layer Scale and Quantity

In the detection framework of YOLO-series models, the multi-scale detection layers P3, P4 and P5 are responsible for object detection tasks of small, medium and large scales, respectively. Statistical analysis of UAV aerial images reveals that the pixel size of the vast majority of objects in this dataset is less than or equal to 20 × 20. In view of this, this section carries out targeted improvement explorations on the number and scale configuration of YOLO detection layers combined with the object size distribution characteristics of the dataset, where √ denotes the addition of the module (the same below).

As shown in Table 3, in the comparative experiments for detection layer configuration, removing the P5 detection head dedicated to large-object detection has a relatively limited impact on the overall model performance. For instance, in the comparison between Experiment (c) and Experiment (d), the recall rate is slightly increased by 0.004 after removing P5, while AP_50_ is slightly decreased by 0.001, with both fluctuations within a small range. This indicates that the contribution of the P5 detection head is relatively low in the infrared dataset of photovoltaic modules adopted in this study. The main reason is that the objects in the dataset are dominated by ultra-small sizes, and large-size objects appear extremely infrequently, so the P5 layer for large-object perception plays a marginal role. To investigate the influence of detection layer scales on model performance, this study further introduces higher-resolution detection layers for evaluation. Experimental results demonstrate that after adding the P2 (160 × 160) detection layer, the model detection performance is significantly improved. Compared with the baseline experiment (a), AP_50_ and AP are increased by 0.072 and 0.03, respectively. On this basis, the P1 (320 × 320) detection layer is further incorporated, and the two metrics are further increased by 0.016 and 0.019, respectively. To achieve a better trade-off between model accuracy and computational efficiency, this study further removes the P5 detection head on the basis of the configuration containing P1 and P2. Experimental results show that the detection layer structure from P1 to P4, as shown in Experiment (f), achieves a remarkable improvement in detection accuracy while maintaining high inference efficiency. Its AP_50_ and AP are increased by 0.097 and 0.053, respectively, compared with the original P3-to-P5 structure. The number of model parameters and model size are reduced by 1.4 M and 2.0 MB, respectively, relative to the original structure.

### 3.5. Ablation Experiment

To investigate the effects of the introduced Dy-C3k2 module, CG-Down downsampling strategy, detection layer adjustment method, and LSDECD detection head on photovoltaic module hotspot detection performance, ablation experiments are conducted based on the constructed UAV aerial dataset, where ODL (optimized detection layer) denotes the optimization strategy for detection layers. The detailed ablation experimental results are presented in Table 4.

Ablation experiments for the infrared detection of hotspots on photovoltaic modules are performed with YOLO11n as the baseline model. When only the baseline model is adopted, its performance metrics are 0.647 for precision (P), 0.630 for AP_50_, and 0.257 for AP, with 2.6 M parameters and a model file size of 5.2 MB. When each improved module is individually introduced on the basis of the baseline, the incorporation of the Dy-C3k2 module increases P to 0.674 and AP_50_ to 0.640, demonstrating its enhancement effect on the adaptability of feature extraction. After introducing the CG-Down module, AP_50_ is improved to 0.650, which reflects the ability of context-guided downsampling to preserve contextual feature information. The performance gain brought by ODL adjustment is the most significant: it not only elevates P, AP_50_, and AP to 0.699, 0.727, and 0.310, respectively, but also reduces the model parameters to 1.2 M and the model size to 3.2 MB, verifying the adaptability of the detection layer scale-adjustment strategy to the target task. With the introduction of the LSDECD detection head, the model performance fluctuates slightly (P drops marginally to 0.645), while AP_50_ increases to 0.643, and the parameters and model size are reduced by 0.3 M and 0.2 MB, respectively. The synergistic effect of multi-module combination is more prominent. The combination of LSDECD and Dy-C3k2 increases P to 0.658 and AP_50_ to 0.651, reflecting the synergistic effect of enhanced feature extraction adaptability and detection head optimization in feature adaptation. When LSDECD, CG-Down, and Dy-C3k2 are combined, P and AP_50_ are improved to 0.675 and 0.654, respectively, indicating better synergy when the three improvements work together. When all improvements are integrated simultaneously, P is increased to 0.723, and AP_50_ and AP are further elevated to 0.730 and 0.315, representing improvements of 0.1 and 0.058 compared with the baseline model, respectively. The model parameters are compressed to 1.8 M, and the model size is reduced to 4.8 MB, which demonstrates that each module can form complementary synergy in feature processing, detection layer design, and detection head enhancement, optimizing the comprehensive detection performance while maintaining the lightweight characteristic of the model.

**Figure 11:**
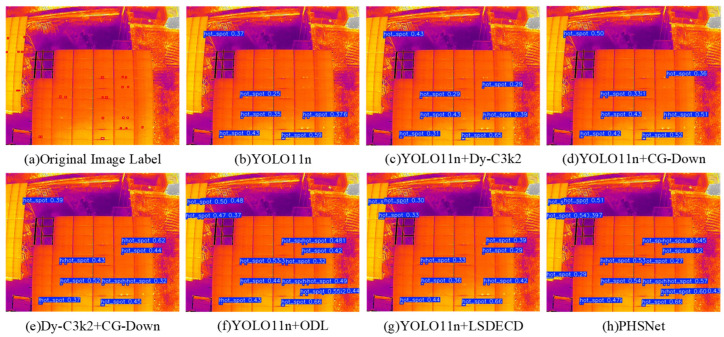
Comparison of visualization results of hotspot images.

Furthermore, to deeply explore the recognition mechanism of the model for hotspot feature information and clarify the differences in detection performance caused by different improvement strategies, several groups of ablation experiment samples are selected for visualization analysis of detection results. The selected images cover multiple hotspot phenomena, and the model can realize target recognition by capturing hotspot features. The detection results are illustrated in Figure 11. It can be observed from the figure that the confidence scores of the target boxes of the PHSNet algorithm are overall superior to those of other models, with a lower miss rate. In addition, PHSNet captures the morphology of hotspots more comprehensively and exhibits higher sensitivity and insight in feature information perception.

### 3.6. Verification of Training Stability and Robustness of PHSNet

**Figure 12:**
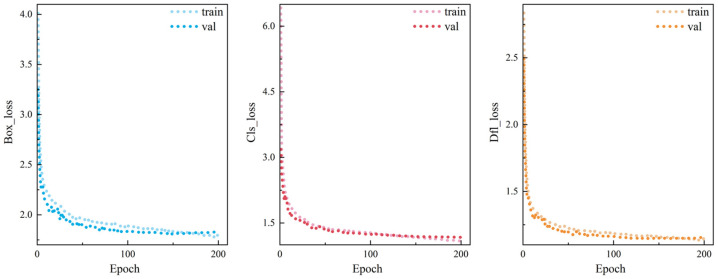
Loss curves in the training process for the PHSNet model.

As can be observed from Figure 12, the proposed PHSNet algorithm presents excellent convergence behavior and training stability throughout the training process. In the training phase, the three core loss functions of the training set, i.e., bounding box regression loss (Box_loss), classification loss (Cls_loss), and Distribution Focal Loss (Dfl_loss), exhibit a gradual decreasing trend with the increase in training iterations. The overall variation is featured by rapid decay at the early stage and gradual stabilization in the later period, which fully validates the effectiveness of the feature learning mechanism of the algorithm. In terms of the loss variation tendency on the validation set, the curves of Box_loss, Cls_loss, and Dfl_loss are highly consistent with those on the training set, indicating that the algorithm does not suffer from overfitting and achieves favorable generalization performance. The above results demonstrate that the algorithm converges steadily during the whole training procedure. The synchronous decline in training loss and validation loss not only confirms the robust generalization ability of the algorithm, but also verifies its strong robustness in the task of infrared hotspot detection for photovoltaic modules.

To verify the anti-noise robustness of the proposed algorithm, Gaussian noise and salt-and-pepper noise are randomly added to the test set images to simulate the imaging degradation of UAV-mounted infrared cameras. The results in Table 5 show that after adding noises, the AP_50_ of YOLO11n decreases from 0.630 to 0.582, a drop of 7.6%. In contrast, the AP_50_ of PHSNet only declines from 0.730 to 0.688, for a reduction of merely 5.8%. Furthermore, PHSNet exhibits a smaller AP degradation compared with YOLO11n. The experimental results demonstrate that in benefiting from the context-guided downsampling module and dynamic convolution feature enhancement module, PHSNet achieves superior anti-noise robustness and feature retention capability for tiny hotspot targets under infrared imaging degradation.

### 3.7. Comparative Experiments with Different Object Detection Models

To systematically validate the comprehensive performance of the proposed PHSNet algorithm in the infrared hotspot detection task for photovoltaic modules, mainstream lightweight YOLO-series object detection models and medium-scale DETR-based models are selected as comparison benchmarks for comparative experiments. To guarantee the fairness of comparison, all models are trained and tested on the same dataset within identical hardware and software environments. As presented in Table 6, PHSNet achieves the best overall performance on this detection task. In terms of detection accuracy, its AP_50_, AP and precision reach 0.730, 0.315 and 0.723, respectively, which are remarkably superior compared to mainstream lightweight detectors including YOLOv8n, YOLOv9-Tiny, YOLOv10n and YOLO11n, as well as the medium-scale RT-DETR-R18 model. In terms of lightweight design and real-time performance, PHSNet only has 1.8 M parameters and a model size of 4.8 MB, which are far lower than those of the RT-DETR series. Under the standard inference setting with a batch size of 1, the inference GPU memory occupation of PHSNet is 216.6 MB, which is higher than that of lightweight YOLO-series models. When the batch size is set to 4, the inference speed reaches 98.8 FPS with an inference latency of 40.5 ms. Although such indicators are slightly inferior to those of some lightweight YOLO variants, they can fully satisfy the real-time detection requirements in practical engineering scenarios.

The proposed PHSNet has only 1.8 M parameters, which belongs to the lightweight level in the general field of object detection. In general, the industry threshold of lightweight detection models is below 5 M. Meanwhile, it is also significantly lighter than all comparison models including YOLO11n and RT-DETR-R18. Although the FPS, inference latency and GPU memory of PHSNet are inferior to those of the baseline YOLO11n, this is a reasonable accuracy–efficiency trade-off. To improve the detection accuracy for ultra-small hotspots (<20 pixels), we add high-resolution detection layers (P1/P2) and context-aware modules, which bring a small loss in efficiency but a significant improvement in precision. Moreover, the inference speed of 98.8 FPS still fully meets the real-time detection requirements of UAV photovoltaic inspection.

**Figure 13:**
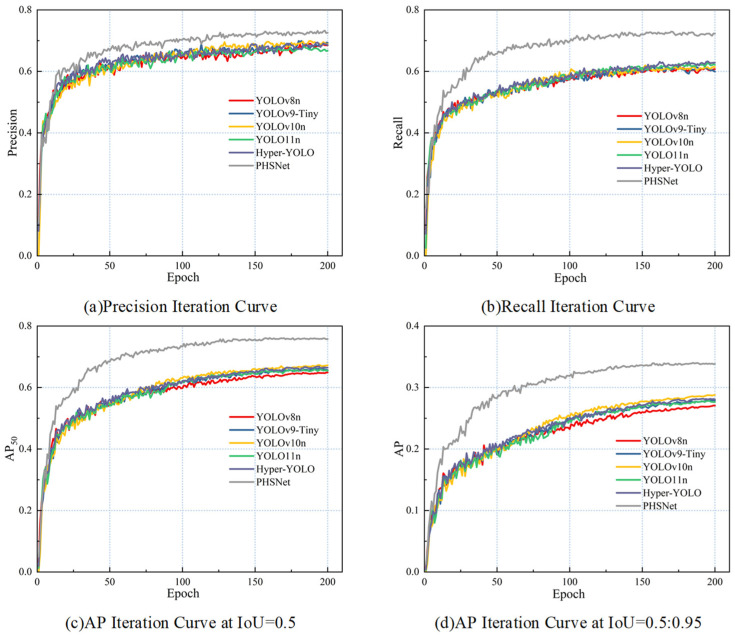
Accuracy curves in the training process of YOLO series models.

Figure 13 plots the iteration curves of four key accuracy metrics—precision, recall, AP_50_, and AP—for PHSNet, YOLOv8n, YOLOv9-Tiny, YOLOv10n, YOLO11n, and Hyper-YOLO during training. In the early training stage (epochs < 50), all metric curves of PHSNet rise at a faster rate. After convergence in the later stage, its performance metrics are distinctly higher than those of the other comparison models. This observation reflects the fast convergence behavior of PHSNet and validates the effectiveness of the proposed YOLO11n-based improvements for infrared hotspot detection in photovoltaic modules.

### 3.8. Visual Verification of Detection Results

**Figure 14:**
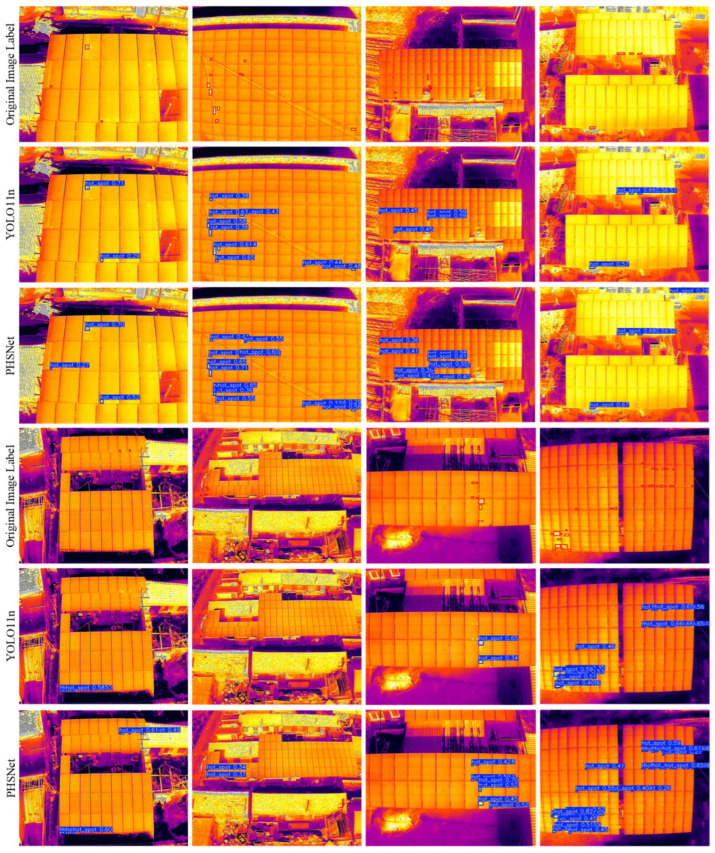
Visualization of detection performance of YOLO11n and PHSNet.

In the visualization experiments of infrared hotspot detection for photovoltaic modules, this paper compares the detection performance of the baseline model YOLO11n and the improved PHSNet. Taking the ground truth bounding boxes of the original images as the reference, the output results for the two models are compared under infrared thermal imaging scenarios of photovoltaic arrays with various layout arrangements, as shown in Figure 14. In terms of detection confidence, compared with the ground truth labels, PHSNet presents a more concentrated confidence distribution and higher overall confidence values for hotspot prediction. Compared with YOLO11n, the confidence scores of the predicted bounding boxes fluctuate less, which effectively reduces mismatches caused by low confidence. In terms of missed detection suppression, when facing complex scenarios with multi-module splicing and strong background interference, YOLO11n is prone to missing tiny hotspots and those located at the edges of photovoltaic modules, according to the ground truth annotations. In contrast, PHSNet achieves more comprehensive detection coverage and higher consistency with the ground truth, significantly reducing the number of missed targets. It should be pointed out that PHSNet still suffers from certain false detections and missed detections. For example, in extreme scenarios with dense hotspots or ultra-low contrast, the model may generate occasional false alarms or fail to detect all real hotspots. This remains a worthy direction for further optimization in future research. Overall, based on systematic comparison with ground truth annotations, PHSNet outperforms the original YOLO11n in both detection confidence stability and missed detection suppression capability for infrared hotspot detection in photovoltaic modules.

### 3.9. Cross-Dataset Generalization Experiment

In this paper, the publicly available Det-Fly dataset [40] is adopted as the data source for the generalization experiment. Oriented to low-altitude small drone detection, this dataset contains more than 13,000 high-resolution images with a resolution of 3840 × 2160 pixels. It covers four typical complex scenarios, including sky, urban, field and mountain, with each scenario accounting for approximately 20–30% of the total data. Notably, around 50% of the drone targets occupy less than 5% of the entire image area, which makes this task a typical small object detection problem. To improve the training efficiency, a total of 8650 images are selected from the Det-Fly dataset for the generalization experiment and divided into training set, validation set and test set at a strict ratio of 7:2:1. To ensure a fair comparison, all training configurations, including hardware setup, hyperparameters and optimization strategies, are kept completely consistent with those in the original photovoltaic hot spot detection experiment. Table 7 summarizes the generalization performance of the baseline and the proposed model on the public drone detection dataset. The proposed PHSNet achieves substantial improvements over YOLO11n across all key evaluation metrics. Specifically, AP_50_ increases by 9.58% (from 0.856 to 0.938), and AP rises by 16.57% (from 0.513 to 0.598). The results verify that the presented model maintains outstanding detection capability in cross-dataset generalization and exhibits stronger robustness for small objects and targets under complex backgrounds.

**Figure 15:**
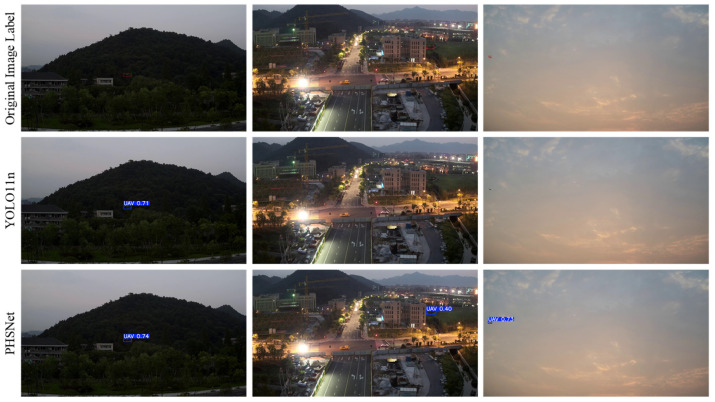
Visualization results on Det-Fly dataset.

The comparison on the detection performance of the two models is shown in Figure 15. In low-light night scenes and scenarios with strong background interference, YOLO11n suffers from missed detections, and PHSNet successfully identifies the targets. In conventional low-contrast scenarios, PHSNet also achieves slightly higher detection confidence than the baseline model. The results indicate that PHSNet possesses stronger robustness in challenging scenarios and superior cross-dataset generalization capability.

## 4. Discussion

Aiming at the core technical challenges of ultra-small target feature loss, low detection accuracy, high miss rate, and the difficult trade-off between lightweight design and detection performance for infrared photovoltaic (PV) hotspot detection in UAV remote-sensing scenarios, this study proposes PHSNet, a lightweight detection network based on the YOLO11n baseline. PHSNet achieves a favorable balance among detection precision, model lightweightness, and real-time inference speed on the dedicated UAV PV infrared hotspot dataset, providing an efficient technical solution for intelligent operation and maintenance (O&M) of large-scale PV power plants. This section provides an in-depth analysis of the internal mechanism of the proposed method, its comparative advantages over state-of-the-art algorithms, engineering application value, generalization capability, typical model failure cases, and research limitations.

First, the multi-module collaborative optimization architecture of PHSNet, which is constructed for feature enhancement, feature preservation, scale adaptation, and lightweight design, systematically solves the core difficulties existing in current PV hotspot detection research. Compared with mainstream algorithms in recent years, PHSNet shows clear advantages. Transformer-based detection models such as FSC-Net [28] and LWMF-DETR [29] improve small-target detection accuracy via attention mechanisms, but they generally suffer from high model complexity and large parameter sizes (about 19.9 M for RT-DETR-R18), which makes them difficult to adapt to edge-deployment environments with limited computing power on UAVs. For lightweight improvements based on the YOLO series, the schemes proposed by Liu et al. [30] and Hao et al. [31] only focus on single-module optimization, and ignore the extreme distribution with over 90% of hotspot targets being smaller than 20 pixels and the irreversible feature loss during continuous downsampling, resulting in insufficient adaptability to aerial infrared scenes. In addition, the enhanced U-Net segmentation method proposed by Awedat et al. [32] achieves high fault segmentation accuracy but relies on pixel-level annotations and offers low efficiency in real-time detection. Different from the above-mentioned methods, PHSNet comprises a well-optimized integration of “feature extraction–feature preservation–scale adaptation–detection output” through the collaboration of Dy-C3k2, CG-Down, optimized detection layers (ODL), and LSDECD, which comprehensively enhances tiny hotspot features and maintains model lightweightness.

Among all improvements, the optimization of detection layer scale and quantity (ODL) yields the most significant performance gain. This design abandons the COCO-defined small-target threshold (32 × 32 pixels), which is severely mismatched with our data distribution, adds high-resolution P1/P2 detection layers, and removes the redundant P5 layer for large objects. This targeted adjustment increases AP_50_ by 0.097 and reduces parameters by 1.4 M compared with the baseline, proving that detection-layer design matched to target-scale distribution is critical for improving ultra-small target detection performance, and provides a reference paradigm for similar aerial remote-sensing detection tasks [33,35].

Second, PHSNet has significant engineering application value. In terms of detection accuracy, its AP_50_ and AP reach 0.730 and 0.315, respectively, outperforming mainstream lightweight YOLO models and the medium-scale RT-DETR-R18. With only 1.8 M parameters and a 4.8 MB model size, it imposes low requirements on the storage and computing power of UAV edge devices. The inference speed of 98.8 FPS and latency of 40.5 ms fully meet the real-time detection needs of UAV aerial inspection, avoiding massive data transmission between UAV and ground station, and greatly reducing the O&M cost of PV power plants.

Third, generalization is verified from two perspectives: internal generalization and cross-dataset generalization. On the one hand, the synchronized decline in training and validation losses indicates good internal generalization without overfitting on the PV hotspot dataset. On the other hand, cross-dataset experiments on the public Det-Fly small-UAV detection dataset show that PHSNet improves AP50 by 9.58% and AP by 16.57% compared with YOLO11n, which validates its reasonable generalization in generic small-target detection scenarios. However, PHSNet still has clear limitations in generalization, that is, the dataset is collected only under clear weather and normal illumination, lacking samples from extreme environments such as heavy fog, strong backlight, rain, and snow; the model uses only single-modal infrared data without fusing visible-light texture information, which limits its ability to distinguish hotspots from structural interference; cross-dataset validation is only performed on the Det-Fly dataset, and more PV-related infrared small-target datasets are needed for further verification. These factors restrict the model’s generalization in complex and diverse scenarios.

Fourth, typical model failure cases and limitations are analyzed in detail. From detection results, we summarize four typical failure scenarios of PHSNet:

(1) Densely distributed hotspots: When multiple tiny hotspots overlap densely, the model tends to miss targets or merge adjacent boxes, due to blurred boundaries of overlapping ultra-small targets and insufficient instance discrimination.
(2) Strong noise interference: Under heavy Gaussian or salt-and-pepper noises, low-contrast hotspots are easily submerged, weakening the feature-enhancement effects of Dy-C3k2 and CG-Down.
(3) Edge-region hotspots: Hotspots at the edges of PV modules or image boundaries are prone to miss detection, caused by receptive-field bias and boundary-padding effects during downsampling.
(4) Extremely low-contrast hotspots: In weak thermal-radiation scenes, the model struggles to stably capture weak edge and gradient features, leading to reduced detection confidence.

In addition to the above failure scenarios, PHSNet has three limitations:

(1) Insufficient environmental robustness: The lack of extreme-weather samples may lead to performance degradation under harsh conditions.
(2) Weak discrimination for dense small targets: No dedicated design for dense targets results in a high miss rate in dense hotspot scenes.
(3) Single-modal limitation: Relying only on infrared data prevents fusing visible-light structural information, leading to occasional false alarms caused by occlusion or background interference.

## 5. Conclusions

Aiming at the problems of fragile feature loss, low accuracy and high miss rates for ultra-small PV hotspots in UAV infrared images, as well as the trade-off between lightweight design and detection performance, this paper proposes PHSNet, a lightweight detection network based on YOLO11n. With four core designs including Dy-C3k2, CG-Down, scale-adaptive detection layers and LSDECD head, PHSNet realizes full-link feature enhancement and lightweight optimization for tiny hotspot targets. Experiments on the UAV PV infrared dataset (92% targets < 20 pixels) show that PHSNet achieves 0.730 AP_50_ and 0.315 AP with only 1.8 M parameters, outperforming YOLO11n by 0.1 and 0.058, respectively. It also exceeds mainstream lightweight detectors and meets the real-time requirement of engineering applications. The consistent loss decline and cross-dataset verification prove its favorable generalization ability for small-target detection. Nevertheless, this study has limitations in environmental robustness, dense small-target discrimination and single-modal dependence. Future work will focus on multi-modal fusion, dense-target optimization, dataset expansion and model compression for better edge deployment. PHSNet provides an efficient solution for real-time intelligent detection of PV hotspots in UAV-based inspections.

## Figures and Tables

**Table 1:** Comparison of multi-scale detection layer parameters in YOLO models.

Detection Layer	Resolution	Downsampling Factor	Corresponding Region on Original Image
P1 (Added)	320 × 320	2x	2 × 2 pixels
P2 (Added)	160 × 160	4x	4 × 4 pixels
P3 (Original)	80 × 80	8x	8 × 8 pixels
P4 (Original)	40 × 40	16x	16 × 16 pixels
P5 (Removed)	20 × 20	32x	32 × 32 pixels

**Table 2:** Experimental parameters.

Parameter Name	Configuration
Training Epochs	200
Batch Size	4
Number of Threads	4
Optimizer	SGD
Initial Learning Rate	0.01
Learning Rate Momentum	0.937
Weight Decay Coefficient Bounding Box Regression Loss Classification Loss	0.0005 CIoU Loss + Distribution Focal Loss (DFL) BCE Loss

**Table 3:** Experimental results of detection layer size and quantity adjustment.

Experimental Group	P1	P2	P3	P4	P5	R	AP_50_	AP	Params (M)	Size (MB)
(a)			√	√	√	0.606 ± 0.013	0.630 ± 0.004	0.257 ± 0.002	2.6	5.2
(b)			√	√		0.618 ± 0.008	0.631 ± 0.003	0.262 ± 0.002	**1.0**	**2.2**
(c)		√	√	√	√	0.689 ± 0.018	0.702 ± 0.003	0.287 ± 0.001	2.7	5.6
(d)		√	√	√		0.693 ± 0.012	0.701 ± 0.007	0.287 ± 0.004	1.1	2.5
(e)	√	√	√	√	√	0.695 ± 0.009	0.718 ± 0.002	0.306 ± 0.002	2.7	6.2
(f)	√	√	√	√		**0.704** ± **0.015**	**0.727** ± **0.004**	**0.310** ± **0.001**	1.2	3.2

Note: The experimental results are reported as mean ± standard deviation over three independent training runs with different random seeds, and the best performance is highlighted in bold. √ indicates that the corresponding module is included in the model.

**Table 4:** Ablation experiment results.

YOLO11n	Dy-C3k2	CG-Down	ODL	LSDECD	P	AP_50_	AP	Params (M)	Size (MB)
√					0.647 ± 0.017	0.630 ± 0.004	0.257 ± 0.002	2.6	5.2
√	√				0.674 ± 0.011	0.640 ± 0.003	0.265 ± 0.003	3.5	6.9
√		√			0.661 ± 0.013	0.650 ± 0.006	0.267 ± 0.005	3.5	7.1
√			√		0.699 ± 0.010	0.727 ± 0.004	0.310 ± 0.001	**1.2**	**3.2**
√				√	0.645 ± 0.018	0.643 ± 0.001	0.263 ± 0.000	2.3	5.0
√	√			√	0.658 ± 0.007	0.651 ± 0.003	0.272 ± 0.001	3.1	6.6
√		√		√	0.673 ± 0.015	0.645 ± 0.004	0.264 ± 0.004	3.2	6.8
√	√	√		√	0.675 ± 0.021	0.654 ± 0.003	0.266 ± 0.003	4.1	8.5
√	√	√	√	√	**0.723** ± **0.016**	**0.730** ± **0.001**	**0.315** ± **0.000**	1.8	4.8

Note: The experimental results are reported as mean ± standard deviation over three independent training runs with different random seeds, and the best performance is highlighted in bold. √ indicates that the corresponding module is included in the model.

**Table 5:** Comparison of model anti-noise performance.

Model	Noise Type	P	R	AP_50_	AP
YOLO11n	Clean Image	0.647	0.606	0.630	0.257
YOLO11n	Noisy Image	0.605	0.558	0.582	0.228
PHSNet (ours)	Clean Image	0.723	0.690	0.730	0.315
PHSNet (ours)	Noisy Image	0.685	0.649	0.688	0.289

**Table 6:** Experimental results of comparison between different models.

Network Model	P	R	AP_50_	AP	Params (M)	Size (MB)	Memory (MB)	FPS	Latency (ms)
YOLOv8n	0.621	0.614	0.615	0.252	2.7	5.4	34.0	**334.5**	**12.0**
YOLOv9-Tiny	0.662	0.605	0.630	0.261	**1.7**	**4.0**	**33.9**	218.3	18.3
YOLOv10n	0.652	0.605	0.637	0.263	2.3	5.5	66.1	223.8	17.9
YOLO11n	0.647	0.606	0.630	0.257	2.6	5.2	67.6	289.7	13.8
Hyper-YOLO	0.681	0.605	0.643	0.264	3.6	7.3	99.7	226.9	17.6
RT-DETR-R18	0.719	0.710	0.711	0.294	19.9	76.9	169.5	109.7	36.5
RT-DETR-R18 (+P2)	0.712	**0.715**	0.717	0.303	18.6	72.4	250.8	71.2	56.2
PHSNet (ours)	**0.723**	0.690	**0.730**	**0.315**	1.8	4.8	216.6	98.8	40.5

Note: The optimal values for each metric in the table are indicated in bold font.

**Table 7:** Generalization experimental results.

Network Model	P	R	AP_50_	AP	Params (M)	Size (MB)	FPS
YOLO11n	0.916	0.793	0.856	0.513	2.6	5.2	**262.9**
PHSNet (ours)	**0.951**	**0.883**	**0.938**	**0.598**	**1.8**	**4.6**	135.7

Note: The optimal values for each metric in the table are indicated in bold font.

## Data Availability

The original data presented in the study are openly available in the PV-HSD-2025 dataset at https://gitee.com/liuyuanlin6445/pv-hsd-2025 (accessed on 1 December 2025).
